# Dispensable Role of Mitochondrial Fission Protein 1 (Fis1) in the Erythrocytic Development of Plasmodium falciparum

**DOI:** 10.1128/mSphere.00579-20

**Published:** 2020-09-23

**Authors:** Mulaka Maruthi, Liqin Ling, Jing Zhou, Hangjun Ke

**Affiliations:** a Department of Microbiology and Immunology, Drexel University College of Medicine, Philadelphia, Pennsylvania, USA; b Department of Laboratory Medicine, West China Hospital, Sichuan University, Chengdu, China; University at Buffalo

**Keywords:** Apicomplexa, Fis1, PfFis1, *Plasmodium falciparum*, malaria, malaria parasite, mitochondrial fission, mitochondrion

## Abstract

Malaria remains a huge global health burden, and control of this disease has run into a severe bottleneck. To defeat malaria and reach the goal of eradication, a deep understanding of the parasite biology is urgently needed. The mitochondrion of the malaria parasite is essential throughout the parasite’s life cycle and has been validated as a clinical drug target. In the asexual development of *Plasmodium* spp., the single mitochondrion grows from a small tubular structure to a complex branched network. This branched mitochondrion is divided at the end of schizogony when 8 to 32 daughter cells are produced, distributing one mitochondrion to each forming merozoite. In mosquito and liver stages, the giant mitochondrial network is split into thousands of pieces and daughter mitochondria are segregated into individual progeny. Despite the significance of mitochondrial fission in *Plasmodium*, the underlying mechanism is largely unknown. Studies of mitochondrial fission in model eukaryotes have revealed that several mitochondrial fission adaptor proteins are involved in recruiting dynamin GTPases to physically split mitochondrial membranes. Apicomplexan parasites, however, share no identifiable homologs of mitochondrial fission adaptor proteins with yeast or humans, except for Fis1. Here, we investigated the localization and essentiality of the Fis1 homolog in Plasmodium falciparum, PfFis1 (PF3D7_1325600), during the asexual life cycle. We found that PfFis1 requires an intact C terminus for mitochondrial localization but is not essential for parasite development or mitochondrial fission. The dispensable role of PfFis1 indicates that *Plasmodium* contains additional fission adaptor proteins on the mitochondrial outer membrane that could be essential for mitochondrial fission.

**IMPORTANCE** Malaria is responsible for over 230 million clinical cases and ∼half a million deaths each year. The single mitochondrion of the malaria parasite functions as a metabolic hub throughout the parasite’s developmental cycle (DC) and also as a source of ATP in certain stages. To pass on its essential functions, the parasite’s mitochondrion needs to be properly divided and segregated into all progeny during cell division via a process termed mitochondrial fission. Due to the divergent nature of *Plasmodium* spp., the molecular players involved in mitochondrial fission and their mechanisms of action remain largely unknown. Here, we found that the only identifiable mitochondrial fission adaptor protein that is evolutionarily conserved in the Apicomplexan phylum, Fis1, it not essential in P. falciparum asexual stages. Our data suggest that malaria parasites use redundant fission adaptor proteins on the mitochondrial outer membrane to mediate the fission process.

## Abstract (precis)

Malaria is responsible for over 230 million clinical cases and ∼half a million deaths each year. The single mitochondrion of the malaria parasite functions as a metabolic hub throughout the parasite’s developmental cycle (DC) and also as a source of ATP in certain stages. To pass on its essential functions, the parasite’s mitochondrion needs to be properly divided and segregated into all progeny during cell division via a process termed mitochondrial fission. Due to the divergent nature of *Plasmodium* spp., the molecular players involved in mitochondrial fission and their mechanisms of action remain largely unknown. Here, we found that the only identifiable mitochondrial fission adaptor protein that is evolutionarily conserved in the Apicomplexan phylum, Fis1, it not essential in P. falciparum asexual stages. Our data suggest that malaria parasites use redundant fission adaptor proteins on the mitochondrial outer membrane to mediate the fission process.

## OBSERVATION

Malaria is a major cause of human morbidity and mortality globally (1). The clinical symptoms of malaria mainly result from repetitive growth of *Plasmodium* parasites inside the red blood cells (RBCs) and periodic rupture of the infected RBCs. After invasion of a host cell, the parasite undergoes growth and division within the parasitophorous vacuole to generate new infective progeny. Unlike many cells that divide via binary fission, the malaria parasite replicates its nuclear DNA for 3 to 5 rounds without concurrent cytokinesis, resulting in the formation of 8 to 32 daughter cells (merozoites). This unique mechanism of division starts at the very end of the asexual life cycle and is termed schizogony (2). In mosquito and liver stages, the parasite’s nuclear DNA undergoes 13 to 14 rounds of replication without cytokinesis, producing up to 10,000 progeny all at once (3); this process is termed sporogony in the mosquito and exoerythrocytic schizogony in the liver. In coordination with this unique cellular reproduction mechanism (schizogony or sporogony), the single mitochondrion of the malaria parasite also undergoes a process of growth and division. In the asexual blood stages of Plasmodium falciparum, the mitochondrion grows from a single small tubular structure to a large branched network over the 48-h intraerythrocytic developmental cycle (IDC) (4); the network is then divided into 8 to 32 pieces to provide one daughter mitochondrion to each merozoite. Since the mitochondrion is essential for parasite growth and replication and cannot be made *de novo*, the process of mitochondrial fission is critical for the malaria parasite.

Studies on mitochondrial fission in model organisms have suggested that the fission machinery that “splits” mitochondrial membranes involves at least two classes of molecules: mitochondrial fission adaptor proteins located on the mitochondrial outer membrane (MOM) and fission GTPases (mechanoenzymes) that are recruited from the cytosol to the mitochondrial fission sites (5). In mammalian cells, multiple mitochondrial fission adaptor proteins have been identified to recruit the fission GTPase (dynamin related protein 1, Drp1), including Fis1 (mitochondrial fission protein 1), Mff (mitochondrial fission factor), and MiD49 and and MiD51 (mitochondrial dynamics proteins 49 kDa and 51 kDa, respectively) (6). Interestingly, besides Fis1, other mammalian MOM-bound fission adaptor proteins (Mff, MiD49 and MiD51) have not been identified in yeast (Saccharomyces cerevisiae) (7), plants (7), or unicellular protozoans. This suggests that mitochondrial fission adaptor proteins are largely species specific. In budding yeast, Fis1 does not directly bind to the dynamin GTPase (Dmn1) at the fission sites but needs a cytosolic adaptor protein Mdv1 (or its paralog Caf4) (8). In apicomplexan parasites, Fis1 is the only mitochondrial fission protein identifiable via bioinformatics (9); BLAST searches of the malaria parasite genome database (www.PlasmoDB.org) did not find any homologs of other mitochondrial fission adaptor proteins in humans or yeast (Mff, MiD49, MiD51 or Mdv1/Caf4). In addition, Fis1 is extremely highly conserved across various *Plasmodium* species (www.PlasmoDB.org). Overall, Fis1 seems to the only evolutionarily conserved mitochondrial fission adaptor protein present throughout eukaryotic kingdoms.

Most Fis1 homologs are small, tail-anchored transmembrane proteins with ∼150 amino acids; the C terminus contains a transmembrane domain (TMD) for anchoring in the MOM and a short C-terminal sequence (CTS), whereas the N terminus has two tetratricopeptide repeats (TPR1/TPR2) that facilitate protein-protein interactions (10). The hypothetical Fis1 in P. falciparum (Pf3D7_1325600, 141 amino acids [aa]) shares 30% sequence identity with human Fis1. A modeled three-dimensional (3D) structure of PfFis1 generated with I-Tasser software (11) is also superimposable on the human Fis1 crystal structure (PDB, 1PC2) (data not shown). In asexual blood stages, PfFis1 is transcribed at all stages, with peak transcription in the late trophozoite and early schizont stages (12), which is consistent with its expected role in mitochondrial fission. However, it has remained unknown whether Fis1 is essential for mitochondrial fission in malaria parasites. The Fis1 homolog in the rodent malaria parasite Plasmodium berghei was not included in the previous large-scale gene knockout (KO) study carried out in this model species (https://plasmogem.sanger.ac.uk/) (13). Furthermore, the recent PiggyBac mutagenesis survey of P. falciparum was unable to unequivocally assign a phenotype to PfFis1 with statistical confidence due to the short length of the gene (<500 bp) (14).

In this study, we generated a conditional PfFis1 knockdown (KD) line and a PfFis1 KO line via CRISPR/Cas9-mediated genome editing. Primers used to perform these genetic studies are listed in Table 1. In both the KD and KO lines of PfFis1, parasites grew normally without noticeable defects, indicating that PfFis1 is not essential for mitochondrial fission. We also discovered the important role of the PfFis1 CTS in its correct subcellular localization.

**TABLE 1:** Primers used in this study

ID^*a*^	Name	Sequence
P1	PfFis1AvrIIFP	taCCTAGGATGGATAGTCCAGAATTACTTAAAATAG
P2	PfFis1BsiWIRP	taCGTACGAAAATACTTGAAAGATTTAAAAGATAAATATAAAC
P3	Fis1KDgRNA1	CATATTTCATATTAAGTATATAATATTGTTAGTTGCACTCACAGCTTGGTTTCAGAGCTATGCTGGAAAC
P4	Fis1KDgRNA2	CATATTTCATATTAAGTATATAATATTGCATTGATATACAAAAGCTCAGTTTCAGAGCTATGCTGGAAAC
P5	Fis1KOgRNA1	TCATATTAAGTATATAATATTAGCGTAATCAAATTGAGTCTGTTTCAGAGCTATGCTGGA
P6	Fis1KOgRNA2	CATATTAAGTATATAATATTGATATTTTTTTCTGAACGTTCGTTTCAGAGCTATGCTGGA
P7	Fis1KDgRNA1-N20	GTTAGTTGCACTCACAGCTTG
P8	Fis1KDgRNA2-N20	GCATTGATATACAAAAGCTCA
P9	Fis1KOgRNA1-N20	AGCGTAATCAAATTGAGTCT
P10	Fis1KOgRNA2-N21	GATATTTTTTTCTGAACGTTC
P11	N20CheckR	ATATGAATTACAAATATTGCATAAAGA
P12	ReverseOligo	TAGGAAATAATAAAAAAGCACCGACTCG
P13	Fis15′HRFP	tgTCCGGAGAAAATGTTAGTAAATAAAAAAAAAAATAC
P14	Fis15′HRRevApaI	CAGGGCCCTTAAAAATACTTGAAAGATTTAAAAGATA
P15	Fis13′UTRFP	atCTTAAGGAGCATTATAAAAAAATATAAGTGTAACG
P16	Fis13′UTRRP	taTCCGGAGATATCGACGTTCATTTCATCTAATAAAAC
P17	Fis1KO5′HRFP	TCCATGGTGCATGGTATATGAGATCGGTATG
P18	Fis1KO5′HRRP	TGAATTCTCTGGACTATCCATTTTCTGGT
P19	Fis1KO3′HRFP	GACTAGTCGATGCAAGAAATAGTAATGCTTTAG
P20	Fis1KO3′HRRP	ACCGCGGACCGTTGCATATATACAACG
P21	Fis1KD 5′CHK	ctCCATTGCCGTATATGCCACAAAAAAAAGTAATAC
P22	3'TetRCheck	ATATTTCATGTCTCAGTAAAGTCTTTCAATAC
P23	PMG75seqF	CTTTAAATTCATGCAAAAATTTAC
P24	Fis1KD 3′CHK	atCGGCCGCTGTTGAAGGTGAGAACAAGCA
P25	Fis1KO 5′CHK	TCTATCATATACGAGAATTCTTGC
P26	Fis1KO5′HRFP	TCCATGGTGCATGGTATATGAGATCGGTATG
P27	hDHFR-HpaIRev	taGTTAACttaATCATTCTTCTCATATACTTCAAATTTGTAC
P28	hDHFR-NarIFwd	atGGCGCCaaaaATGCATGGTTCGCTAAACTGCATC
P29	PfFis1KO3′CHK	ACTCGCCTTATACATTTAAAGCA
P30	Fis1qRTFp	TCAATTTGATTACGCTTGTTTGTT
P31	Fis1qRTRp	GCATCGATTTTTAATAAGGCATTT
P32	seryl-tRNA synthetase_F	AAGTAGCAGGTCATCGTGGTT
P33	seryl-tRNA synthetase_R	TTCGGCACATTCTTCCATAA

a ID, identifier.

### PfFis1 is localized to the parasite mitochondrion, and a conditional knockdown of PfFis1 does not cause defects in the parasite.

**FIG 1:**
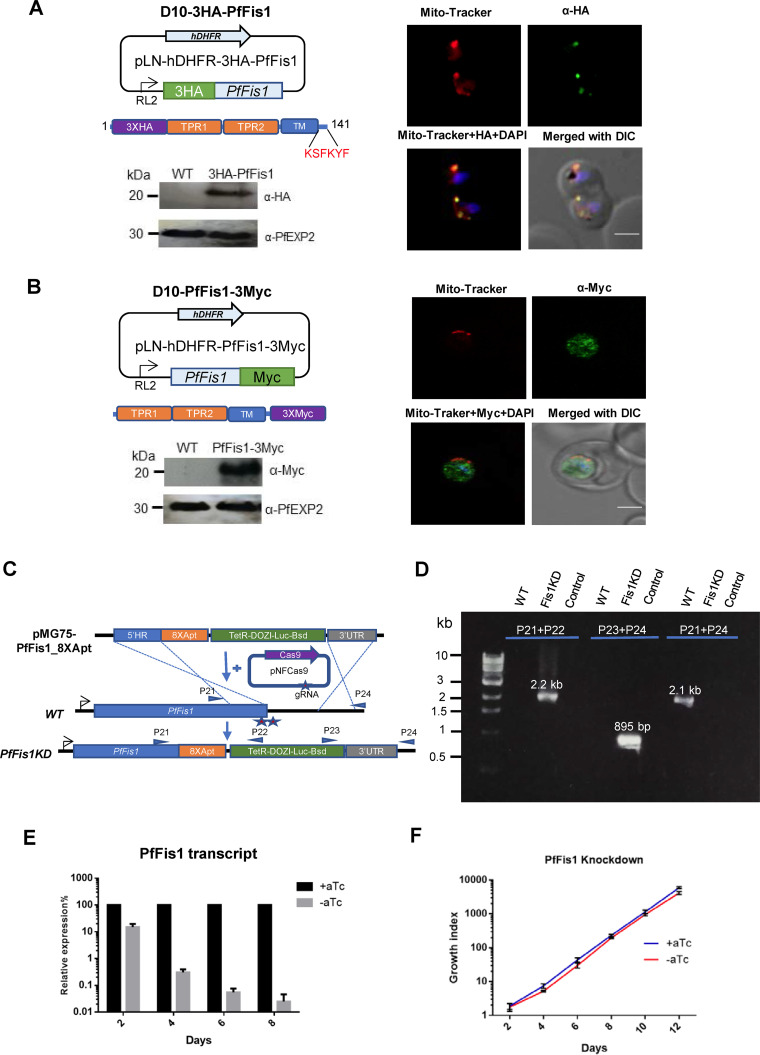
PfFis1 localization with N- or C-terminal tagging and PfFis1 KD without tags. (A) The pLN-based construct used for ectopic expression of N-terminal 3HA-tagged PfFis1. The CTS of PfFis1 (KSFKYF) is highlighted in red. Representative IFA images show localization of 3HA-PfFis1. Parasites were probed with mouse anti-HA (green), MitoTracker (red), and DAPI (4′,6-diamidino-2-phenylindole) (blue) and merged with differential interference contrast (DIC). Scale bar, 2 μm. Western blotting data show the expression of 3HA-PfFis1. Anti-HA and anti-PfEXP2 (loading control) antibodies were used. (B) The pLN-based construct used for ectopic expression of C-terminal 3Myc-tagged PfFis1. Representative IFA images show localization of PfFis1-3Myc. Parasites were probed with mouse anti-Myc (green), MitoTracker (red), and DAPI (blue) and merged with DIC. Scale bar, 2 μm. Western blotting data show the expression of PfFis1-3Myc. Anti-Myc and anti-PfEXP2 (loading control) antibodies were used. (C) CRISPR/Cas9-based system used to integrate the TetR-DOZI-aptamer system at the 3′ end of the endogenous PfFis1 gene. Positions of primers used as described for panel D are highlighted. (D) Diagnostic PCR showing the correct genotype of the PfFis1 KD line after genome editing. The PCR product of primers P21 and P22 shows the 5′ integration; the PCR product of primers P23 and P24 shows the 3′ integration; the PCR product of primers P21 and P24 shows the WT genotype. Primers P21 and P24 failed to amplify any bands from the KD parasites due to the large size of the fragment to be amplified (>11 kb). Control PCRs contained no-template DNA. (E) Analysis of PfFis1 transcripts in the KD parasites via qRT-PCR. At each time point, the PfFis1 transcription level in the aTc-minus culture was compared to that in the aTc-plus culture (the latter was normalized to 100%). Seryl-tRNA synthetase was used as an internal control. Error bars indicate standard deviations of results from triplicate samples; this experiment was repeated two times. (F) Effect of PfFis1 KD on the growth of P. falciparum. To quantify the growth, parasites were subjected to Percoll enrichment, and equal numbers of PfFis1KD parasites were grown in the presence and absence of aTc in the medium (+aTc and −aTc, respectively) for 12 days (6 IDCs). Parasitemia was quantified on alternate days, and the parasitemia value was multiplied by the parasite dilution factor to produce a measure of cumulative growth. All data points represent means ± standard deviations (SD) of results from three independent experiments. TRP1, tetratricopeptide repeat 1; TRP2, tetratricopeptide repeat 2; TM, transmembrane domain.

In order to detect the localization of PfFis1 in P. falciparum, we episomally expressed tagged PfFis1 fusion proteins with small epitopes in D10 wild-type (WT) parasites. In one transgenic line, we tagged PfFis1 with 3× hemagglutinin (3HA) at the N terminus (Fig. 1A). In a second line, PfFis1 was tagged with 3Myc at the C terminus (Fig. 1B). In both parasites lines, episomal expression of PfFis1 was driven by the promoter of a mitochondrial gene, the 5′ untranscribed region (5′-UTR) of the annotated mitochondrial ribosomal protein L2 (PfmtRPL2, PF3D7_1132700) (15). Similarly to PfFis1, PfmtRPL2 exhibits peak transcription at the late trophozoite and schizont stages (www.PlasmoDB.org). Expression of tagged PfFis1 at the predicted molecular weight was confirmed by Western blotting (Fig. 1A and B). To verify the subcellular localization of PfFis1, we performed immunofluorescence assays (IFA) in both transgenic parasites. When PfFis1 was tagged with 3HA at the N terminus, it was localized, as expected, to the mitochondrion (Fig. 1A). However, when tagged with 3Myc at the C terminus, PfFis1 was found dispersed throughout the cytosol (Fig. 1B). Recent investigations in another apicomplexan parasite, Toxoplasma gondii, revealed that truncation of the entire TMD and CTS of Fis1 (TgFis1) resulted in mislocalization of the protein to the cytoplasm (16), whereas removal of the CTS (LSK) alone did not affect Fis1’s localization to the MOM (17). It is interesting that PfFis1 has a longer CTS (KSFKYF) than TgFis1 (LSK) and that this 6-amino-acid CTS is highly conserved across *Plasmodium* species. Taken together, our data suggest that addition of 3Myc sequence to the CTS of PfFis1 blocked proper protein trafficking and localization of PfFis1 to the MOM.

To determine the role of PfFis1 in parasite survival, we first utilized the CRISPR/Cas9-mediated (18) TetR-DOZI-aptamer system (19) to conditionally knock down the endogenous expression of PfFis1. The use of the conditional KD system is beneficial to evaluate the essentiality of malarial genes, since the parasite maintains a haploid genome in the blood stages and live parasites null for an essential gene cannot be obtained. Since our data revealed the importance of PfFis1 CTS at its correct localization, we modified our conventional KD systems (20, 21) to reduce the level of expression of PfFis1; however, no tags were added to its C terminus (Fig. 1C). We cotransfected WT P. falciparum (D10 strain) with the template plasmid, pMG75noP-Fis1-8apt, and with two guide RNA (gRNA) constructs that were designed to guide Cas9 cleavage near the end of the PfFis1 locus. The transfected parasites were selected using media containing blasticidin and anhydrotetracycline (aTc), yielding a conditional KD line. The small molecule aTc maintains the expression level of the target gene by preventing the negative regulator, the TetR-DOZI fusion protein, from binding to the target mRNA via RNA aptamers (19). Hence, expression of PfFis1 was expected to be maintained in aTc-supplemented media but abrogated upon aTc removal. The genotype of the PfFis1 KD line was confirmed by diagnostic PCRs using site-specific primers (Fig. 1D). Since no antibodies were available to detect PfFis1 protein, we verified the KD efficiency by quantitative real-time PCR (qRT-PCR) to quantify PfFis1 mRNA transcripts in parasites upon aTc removal for 2, 4, 6, and 8 days (representing up to 4 IDCs). In comparison to aTc plus controls, the level of PfFis1 transcript dramatically decreased after aTc removal for 1 IDC (2 days) and continued to decrease to a negligible level over the KD time course (Fig. 1E). However, with or without aTc, the parasites did not show any defects in growth rate (Fig. 1F), indicating a dispensable role of PfFis1 in parasite viability. Interestingly, our data also revealed that in the absence of aTc, the TetR-DOZI-aptamer system not only prevents protein translation by pulling the mRNA away from ribosomes (19) but also causes a rapid degradation of the target mRNA. To the best of our knowledge, this is the first report showing that the TetR-DOZI-aptamer system could also interfere with the mRNA stability of the target transcript.

### PfFis1 is dispensable for mitochondrial fission.

**FIG 2:**
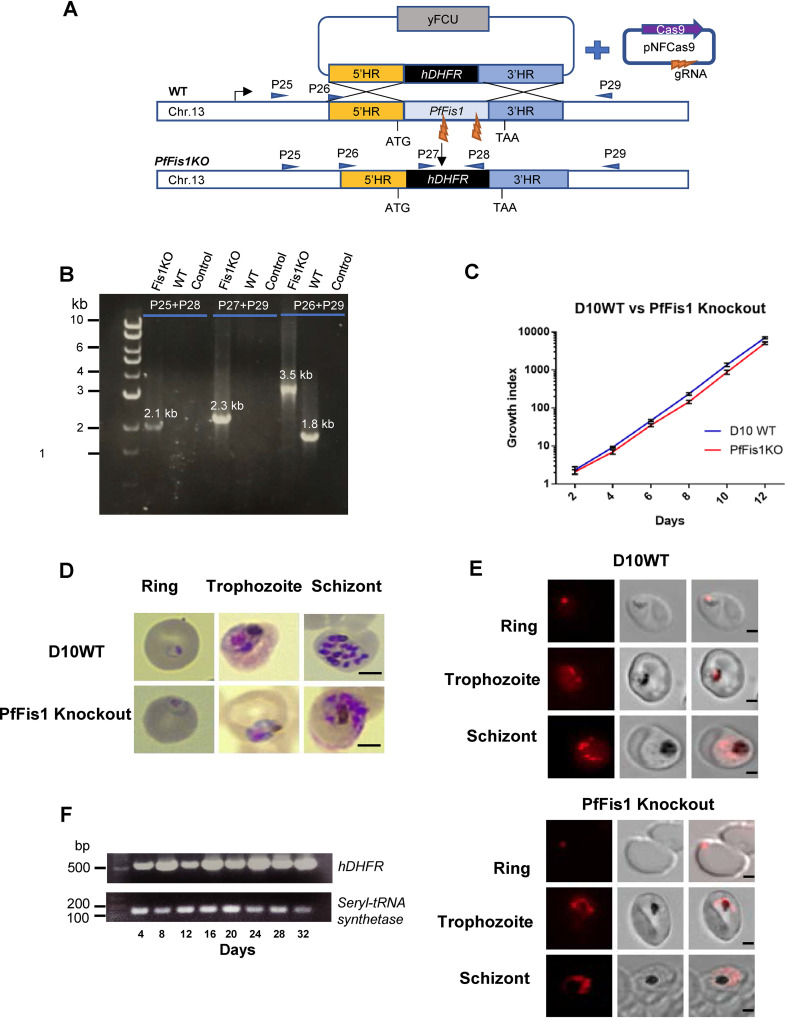
PfFis1KO does not affect mitochondrial fission. (A) A schematic representation showing the CRISPR/Cas9-mediated replacement of PfFis1 open reading frame (ORF) with the hDHFR cassette. Positions of primers used as indicated in panel B are highlighted. (B) Diagnostic PCRs showing the correct genotype of the PfFis1KO line after genome editing. The PCR product of primers P25 and P28 shows 5′ integration; the PCR product of primers P27 and P29 shows 3′ integration; the PCR product of primers P26 and P29 shows the entire locus. The KO band (3.5 kb) is bigger than the WT band (1.8 kb) due to insertion of the hDHFR cassette. (C) Growth curve analysis of PfFis1KO line compared to D10 WT. The two parasite lines were tightly synchronized and diluted to an initial parasitemia level of 1% at ring stages and monitored by analyzing Giemsa-stained blood smears over 12 days. All data points represent means ± SD of results from three independent experiments. (D) Morphology of the PfFis1KO parasites analyzed using Giemsa-stained blood smears. Scale bars equal 2 μm. (E) Live parasite analysis of mitochondrial morphologies. Live PfFis1KO and D10 WT parasites were treated with MitoTracker (10 nM) for 30 min and washed three times. Images were acquired at the ring, trophozoite, and schizont stages. Scale bars equal 2 μm. (F) Growth competition between PfFis1KO and D10 WT parasites. The housekeeping gene seryl-tRNA synthetase was used as an internal control.

Thus far, our data have shown that PfFis1 is likely nonessential for the parasite. To rule out the possibility that a small amount of PfFis1 protein in the KD parasite was sufficient to maintain parasite health, we attempted to knock out PfFis1 via CRISPR/Cas9 genome editing. In D10 WT, we cotransfected the KO template plasmid carrying two homologous sequences of PfFis1 with two gRNA constructs that guide Cas9 cleavage in the middle of the PfFis1 genetic locus (Fig. 2A). Transfected parasites were selected by WR99210, a specific inhibitor of the *Plasmodium* dihydrofolate reductase gene (22), and drug-resistant parasites were obtained. The genotype of the transgenic parasite line was confirmed by diagnostic PCRs using site-specific primers (Fig. 2B). PfFis1 was successfully knocked out, yielding a PfFis1KO line (PfFis1KO). We tightly synchronized the PfFis1KO and WT lines and observed the growth kinetics over 6 IDCs by counting parasitemia in blood smears of each culture. PfFis1KO parasites did not exhibit any noticeable growth defects compared to WT (Fig. 2C), nor did they appear morphologically abnormal (Fig. 2D), suggesting that the complete removal of PfFis1 had not caused problems for parasite growth and replication. To monitor mitochondrial morphology of PfFis1KO parasites, we stained them with a fluorescent dye MitoTracker and performed live-cell microscopy. In comparison to WT controls, the mitochondrion of PfFis1KO displayed normal development during the asexual blood stage; importantly, it underwent fission in the late schizont stage to produce fragmented mitochondria to be distributed into daughter cells (Fig. 2E). In addition, we mixed equal numbers of PfFis1KO and WT parasites in one flask, obtained DNA samples every 2 IDCs (4 days), and detected the presence of PfFis1KO parasites by PCR (Fig. 2F). Only PfFis1KO contained the exogenous hDHFR gene (human dihydrofolate reductase gene, the transfection selectable marker), and the robustness of the PCR band persisted throughout 16 IDCs (32 days), suggesting that there was no or negligible fitness cost associated with complete deletion of PfFis1. Collectively, our data suggest that PfFis1 is not essential for mitochondrial fission in the asexual blood stages of P. falciparum.

### Conclusions.

Our data support the notion that PfFis1 relies on its short C-terminal tail for mitochondrial localization but is not essential for mitochondrial fission or parasite survival in asexual stages of P. falciparum. Our Fis1 KO data in malaria parasites are consistent with the idea of the nonessentiality of Fis1 proposed by KD approaches carried out in Toxoplasma gondii (23). Discovered first in yeast in 2000 (24), Fis1 has been evolutionarily conserved in most eukaryotes that contain mitochondria. In yeast, Fis1 is the only known MOM-bound fission adaptor protein, and it is essential for mitochondrial fission (25). In human cells, the role of Fis1 in recruiting Drp1 is less critical, since several other MOM-bound fission adaptor proteins (Mff, MiD49, MiD51) are capable of fulfilling the same function (6, 26). Interestingly, in parasitic apicomplexan parasites that contain significantly smaller genomes, we and others (16, 23) have suggested that their mitochondria likely harbor additional MOM-bound fission adaptor proteins to mediate mitochondrial fission. Hence, we propose that the mitochondrial fission model in apicomplexan parasites likely resembles that of human cells rather than that of yeast. Further studies are required to identify additional mitochondrial fission adapter proteins in different genera of the Apicomplexan phylum. In particular, any novel and essential mitochondrial fission adaptor protein of apicomplexan parasites would represent a potential antiparasite drug target, as it is likely absent in human mitochondria.

### Primers.

Primers used in this study are listed in Table 1.

### Parasite culture and transfection.

Plasmodium falciparum WT strain D10 was used for all transfections in this study. Parasite culture and transfection procedures followed our previously published protocols (20, 21).

### Plasmid construction.

To tag PfFis1 with 3Myc at the C terminus, the plasmid pLN-hDHFR-PfFis1-3Myc was constructed by amplifying the PfFis1 gene from P. falciparum genomic DNA using primers P1 and P2. The PCR product was digested with AvrII and BsiWI and inserted into the expression vector pLN-hDHFR-3Myc (20). For N-terminal tagging of PfFis1 with 3HA, a synthetic DNA fragment (AvrII-3HA-BsiWI-PfFis1-AflII) was obtained from Genewiz. The sequence of the DNA fragment is as follows: tatttttttttgttaatattatacaatataCCTAGGAAAAATGTATCCATACGACGTTCCTGACTATGCCGGATACCCATACGACGTGCCTGATTACGCCGGTTCTTACCCTTACGACGTTCCTGACTACGCCGCACAACGTACGATGGATAGTCCAGAATTACTTAAAATAGAACTTCAAAGATTAAAGAATGATTATGAAAATGAACTATCAGTAGATCACGTAATGCCCAAGACTCAATTTGATTACGCTTGTTTGTTAATATGTTCTTCAGATTTGAAGAACATAAAGTTCGCTTCTTCATTGTTGCATGAATTGTTGTTCATAAATTATAATCGTATAGATTGTTTATATCAGCTAGCTATAGCACATATAAAATTAAGAGATTATAAAAAAGCTAAGAATTATTTAAATGCCTTATTAAAAATCGATGCAAGAAATAGTAATGCTTTAGCTTTAAAGAGTTTACTTTTTGATTTAATATCATCTGATGGTTTAATTGGTGCTTTGTTAGTTGCACTCACAGCTTGTGGTTTATATTTATCTTTTAAATCTTTCAAGTATTTTTAActtaaggtcgagttatataatatatttatgtactcg.

The first 30 and the last 36 bp of this synthetic DNA fragment (small letters) are homologous to the ends of the pLN-hDHFR-3HA construct present after restriction digestion with AvrII and AflII sites (20). The sequences that are homologous between the synthetic DNA and the digested vector allowed them to be joined by a DNA assembly reaction (NEBuilder; New England Biolabs).

For both CRISPR/Cas9-mediated KD and KO studies, guide RNAs (gRNAs) were identified from the gene sequence using the Eukaryotic Pathogen CRISPR guide RNA design tool (http://grna.ctegd.uga.edu/). They were individually cloned into the NFCas9-yDHOD(-) plasmid, which contains a full-length Cas9 gene from Streptococcus pyogenes without any tags and a gRNA expression cassette (21). The gRNA sequences are listed as P3 to P6. Diagnostic PCRs were used to identify positive-testing clones from DNA assembly reactions. These primers are listed as P7 to P10. The reverse primer P11 was used as previous described (21). All gRNA constructs were sequenced by Genewiz using primer P12.

For constructing the template plasmid for KD studies without tags, the 5′HR (5′ homologous region) of PfFis1 was amplified from genomic DNA by the use of primers P13 and P14. The 3′UTR (3′ homologous region) of PfFis1 was amplified from genomic DNA by the use of primers P15 and P16. The 3′UTR was cloned into the pMG75noP-8apt-3HA vector (21) using AflII and BspEI, whereas the 5′HR fragment was subsequently cloned using BspEI and ApaI, yielding the plasmid pMG75noP-Fis1-8apt for KD. This plasmid was linearized with EcoRV before transfection. For construction of the template plasmid for KO studies, the 5′HR was amplified from genomic DNA by the use of primers P17 and P18. The 3′HR was amplified from genomic DNA by the use of primers P19 and P20. The two HR fragments were sequentially cloned into pCC1, yielding the plasmid pCC1-5′3′Fis1 for KO. This plasmid was linearized with HincII before transfection. Primers P21 to P24 were used to check the genotype of the KD line; primers P25 to P29 were used to check the KO genotype.

### Parasite growth kinetics, IFA, Western blotting, and live MitoTracker staining.

These procedures followed our published protocols (20, 21).

### Nucleic acid extraction, PCR, and qRT-PCR.

Genomic DNA from late-stage parasites was isolated with a DNeasy blood and tissue kit (Qiagen). During the KD time course (days 2, 4, 6, and 8), total RNA from parasites representing each set of conditions (aTc plus versus aTc minus) was isolated from saponin-lysed parasite pellets followed by treatment with TRIzol (Thermo) and purification with an RNeasy kit (Qiagen). After treatment with DNase I (New England Biolabs), 2 μg RNA from each set of conditions was primed with random hexamers and converted to cDNA using SuperScript III reverse transcriptase (Thermo). qRT-PCR was carried out in triplicate with SYBR green real-time PCR master mixes (Thermo) in a real-time PCR instrument (Applied Biosystems). Primers used for amplification of PfFis1 are listed as P30 to P31. A previously reported housekeeping gene, encoding seryl-tRNA synthetase, was used as the internal control (primers P32 to P33) (27). Data were analyzed using threshold cycle (2^−ΔΔ^*^CT^*) method as previously described (28). For regular PCR, a reaction volume of 25 μl was used and the extension temperature of the *Taq* polymerase was kept at 62°C.
